# Rapid and visual differentiation of pseudorabies virus vaccine and wild-type strains using an RPA-LFD assay

**DOI:** 10.1186/s12917-026-05828-7

**Published:** 2026-08-18

**Authors:** Yumei Chen, Xiuli Deng, Yanli Shang, Jifei Yang, Huifang Hao, Shujun Chai, Yunchao Liu

**Affiliations:** 1 School of Life Sciences, Zhengzhou University, Zhengzhou, Henan China; 2 Institute for Animal Health, Key Laboratory of Animal Immunology, Henan Academy of Agricultural Sciences, Zhengzhou, 450002 China

**Keywords:** PRV, *gE*, Recombinase polymerase amplification (RPA), Lateral flow diagnostic strips (LFD)

## Abstract

Pseudorabies virus (PRV) inflicts substantial economic losses on the global swine industry. Accurate differentiation between vaccine-derived and circulating wild-type strains is critical for effective disease control and eradication programs. However, conventional diagnostic methods often rely on costly instrumentation and entail lengthy turnaround times, thereby limiting their utility for rapid, field-deployable differential diagnosis—particularly in resource-constrained settings. In this study, we developed a rapid detection system integrating recombinase polymerase amplification (RPA) with lateral flow diagnostic strips (LFD) for the simultaneous identification of PRV wild-type and vaccine strains. The assay demonstrated high analytical specificity, reliably differentiating between PRV vaccine and wild-type strains, exhibiting no cross-reactivity with other prevalent swine pathogens. It also exhibited high sensitivity, yielding clear visual results within 20 min and achieving a limit of detection of 80 copies/µL for the PRV *gE* gene. Moreover, the method is operationally straightforward, requires minimal training, delivers unambiguous visual readouts, and is highly cost-effective. Collectively, the RPA-LFD platform represents a robust, field-adaptable tool for on-site surveillance of PRV field strains, offering significant potential to support large-scale screening and advance pseudorabies eradication initiatives.

**Supplementary Information:**

The online version contains supplementary material available at https://doi.org/10.1186/s12917-026-05828-7.

## Introduction

*Pseudorabies virus* (PRV), also known as *Aujeszky’s disease virus* or *Suid alphaherpesvirus 1* (*SuHV-1*), is an enveloped double-stranded DNA virus belonging to the genus *Varicellovirus*, subfamily *Alphaherpesvirinae*, family *Herpesviridae* [1, 2]. Pigs are the natural host and reservoir of PRV and are the only species capable of surviving infection [3]. Following PRV infection, clinical symptoms vary markedly among pigs at different growth stages, including reproductive failure in sows, fatal encephalitis with 100% mortality in neonatal piglets, and respiratory distress as well as growth retardation in weaned piglets [4]. Such clinical manifestations cause substantial economic losses to the swine industry in China. Furthermore, several studies have utilized metagenomic next-generation sequencing to identify specific PRV sequences within patients’ tissues [5–10].

Currently, vaccines based on *gE*-deleted PRV strains and their corresponding diagnostic reagents play a crucial role in stabilizing pseudorabies control in swine [11]. Classic attenuated live vaccines such as Bartha K61 have historically been effective, yet emerging novel variant strains in China since 2011 have compromised the protective efficacy of conventional vaccines [12, 13]. Critically, while vaccination can effectively prevent the manifestation of clinical symptoms, it does not eradicate the wild-type PRV present in infected animals, nor does it prevent subsequent infections by wild-type strains. Latently infected pigs may thus act as reservoirs for silent transmission [14]. Therefore, a reliable and practical method to differentiate wild-type PRV infection from *gE*-deleted vaccine immunization is urgently needed for effective surveillance and eradication programs.

Over the past two decades, researchers have established a variety of molecular detection methods targeting the *gE* gene of PRV, including conventional polymerase chain reaction (PCR), TaqMan probe-based real-time quantitative PCR (qPCR), duplex droplet digital PCR (ddPCR), and nanoparticle-assisted PCR (nanoPCR) [15–18]. Although these methods exhibit high analytical sensitivity, their clinical utility remains constrained by practical limitations—most notably the requirement for costly thermal cyclers and highly trained personnel.

In recent years, isothermal amplification technologies have significantly broadened the diagnostic toolkit. In particular, the integration of recombinase polymerase amplification (RPA) with lateral flow dipstick (LFD) detection enables a rapid, instrument-free, and visual readout. An increasing number of studies have described successful applications of (RT-)RPA-LFD for the visual detection of diverse viral pathogens, including canine distemper virus (CDV), tilapia lake virus (TiLV), duck adenovirus type 3 (DAdV-3), dengue virus type 2 (DENV-2), and orthoflaviviruses [19–23]. Collectively, these studies have established a robust methodological foundation for RPA-LFD in viral pathogen diagnostics and provide a strong rationale for extending this approach to field-based surveillance of PRV.

To facilitate rapid on-site detection of both wild-type and vaccine strains of PRV, in this study we established an RPA-LFD assay for the specific detection of PRV. This RPA-LFD platform combines the high sensitivity of isothermal amplification with the simplicity and portability of lateral-flow strips, thereby providing a promising tool for field-based screening and supporting PRV surveillance and control efforts.

## Materials and methods

### Principle of the RPA-LFD

**Fig. 1:**
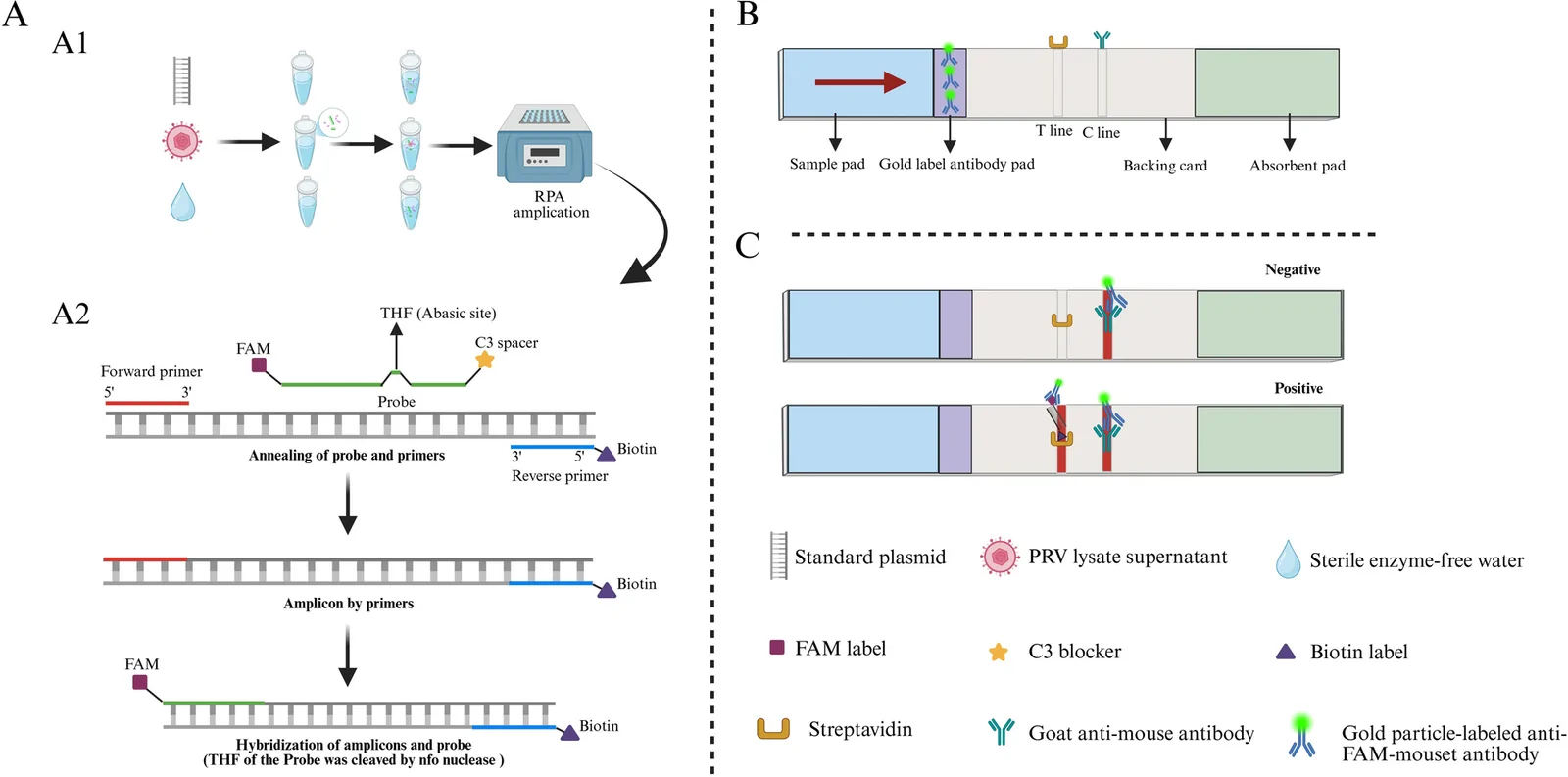
Schematic diagram of RPA-LFD detection of PRV. **A **Workflow of the RPA assay for PRV DNA amplification. A1: Sample preparation and RPA amplification using PRV lysate supernatant, standard plasmid, or sterile water as templates. A2: Principle of RPA with a FAM-labeled probe and biotin-labeled reverse primer, where the THF a basic site in the probe is cleaved by Nfo nuclease during amplification to generate dual-labeled amplicons. **B **Structure of the LFD strip, consisting of a sample pad, a gold-labeled antibody pad, a nitrocellulose membrane with test and control lines, and an absorbent pad, assembled on a backing card. **C** Schematic of LFD results: negative control shows only the C line, while a positive sample displays both T and C lines, indicating successful PRV detection

A schematic of the assay for differentiating PRV vaccine strains from wild-type strains is shown in Fig. 1. The assay workflow seamlessly integrates RPA-mediated nucleic acid amplification with LFD-based visual detection. Briefly, RPA reactions were established using specific primers and a probe designed against the PRV *gE* gene. The reverse primer was modified with biotin at its 5' end, and the probe was labeled with FAM at the 5' end, contained a tetrahydrofuran (THF) abasic site mimic internally, and was blocked with a C3 Spacer at the 3' end. Amplification reactions were performed at a constant temperature of 45 °C for 20 min in a metal bath and generated dual-labeled (biotin and FAM) RPA products. 

For detection, the RPA product is applied to the sample pad of the LFD (Fig. 1B). As the solution migrates via capillary action, FAM-labeled amplicons bind to anti-6-FAM antibodies labeled with gold nanoparticles (AuNPs), forming labeled complexes. These complexes are captured at the test line (T line) by streptavidin via the biotin-streptavidin interaction. Subsequently, excess AuNP-labeled conjugates continue to flow and are captured at the control line (C line) by goat anti-mouse antibodies. The accumulation of AuNPs leads to the formation of a red band that is visible to the naked eye. A valid test requires a visible C line. A positive result (present of the *gE* gene of wild-type PRV) is indicated by both T and C lines, while a negative result (*gE* gene absent, as in vaccine strains or negative samples) is indicated by only the C line.

### Materials and viral strains

The PRV *gE* DNA templates utilized in this experiment were synthesized by Sangon (Shanghai, China). All primers and probes were synthesized by EZassay (Shenzhen, China).

Pseudorabies virus (PRV, HeNLH/2017), porcine circovirus (PCV-2b), and porcine reproductive and respiratory syndrome virus (PRRSV, NADC30-like) were stored in this laboratory. Furthermore, porcine epidemic diarrhea virus (PEDV, ZJ08 strain), porcine transmissible gastroenteritis virus (TGEV, HB08 strain), and classical swine fever virus (CSFV, AV1412) were all acquired from commercially available attenuated live vaccines provided by China Animal Husbandry Industry Co., Ltd. All live virus manipulations were performed in a Biosafety Level 2 (BSL-2) laboratory following standard institutional biosafety protocols.

### Primers and probe design

Primers and probes were designed based on a conserved region of the PRV *gE* gene (GenBank accession number: OR062216.1) using Primer Premier 5.0, following the design guidelines outlined in the Huixin fluorescent isothermal amplification reagent manual. qPCR primers and probes were designed as described by Quan et al. [24]. The sequences are as follows:

Forward primer:

F1: GCTTCCACGCGCTCGGCTTCCACTCGCAGCTCT.

F2: ACTACGTGTACGAGCCCTGCATCTACCACCC.

qF: TTCCACTCGCAGCTCTTCT.

Reverse primer:

R1: GCTCGTACACGTAGTACAGCAGGCACCGCGGGG-Biotin.

R2: ACGTGGCCGTTGTGGGTCATCACGAGCACGTACA-Biotin.

qR: GAGTCGCCCATGTCCGAGA.

Probe:

P1: 5'-FAM-GTGGTCTCGGACATGGGCGACTCGCGCGAGA-THF-CTTTACCGCCACGCTG-C3 Spacer-3'.

P2: 5'-FAM-ACGCCTCGTGCAGCCCGCTGCTCGGGGACCG-THF-TGGCTGACCGCCTGCC-C3 Spacer-3'.

qP: 5'-Cy5-ACACGTTCGACCTGATG-MGB-3'.

### Construction of the RPA, conventional PCR and qPCR systems

RPA was performed as previously described [25] with minor modifications, within a 25 µL reaction system using the Fluorescent Isothermal Amplification Reagents (Hangzhou Perfect mRNA Biotechnology Co., Ltd., Hangzhou, China). Specifically, each reaction mixture comprised 1 µL each of the forward and reverse primers (10 µM), along with 0.3 µL of probe (10 µM), 2 µL of template DNA, and 20.7 µL of DEPC-treated water (Servicebio, G3004-100mL, Wuhan, China). Additionally, a lyophilized enzyme pellet and a magnesium acetate pellet were included in the mixture. The tube was mixed thoroughly and promptly incubated at 45 °C for 20 min.

Conventional PCR was carried out in a 50 µL system using Premix Taq (RR902A, Takara, Kusatsu, Shiga, Japan) according to the manufacturer’s instructions, based on the protocol reported by Saiki et al. [26]. The reaction mixture included 25 µL Ex Taq premix, 2 µL template DNA, 0.5 µL forward primer (10 µM), 0.5 µL reverse primer (10 µM), and 22 µL DEPC-treated water. The PCR thermal profile was as follows: initial denaturation at 98 ℃ for 2 min; 35 cycles of 98 ℃ for 10 s, 52 ℃ for 30 s, 72 ℃ for 20 s; final extension at 72 ℃ for 1 min.

qPCR was performed according to the method described by Quan et al. [24], with a 25 µL reaction system prepared using Animal Detection U+ Probe qPCR Super PreMix (QV114-01, Vazyme, Nanjing, Jiangsu, China). Each reaction consisted of 12.5 µL qPCR Super PreMix, 2 µL template DNA, 0.15 µL forward primer (10 µM), 0.15 µL reverse primer (10 µM), 0.05 µL probe (10 µM), 0.5 µL 50× ROX reference dye, and 9.65 µL DEPC-treated water. The qPCR amplification program was set as: 37 ℃ for 2 min, initial denaturation at 95 ℃ for 30 s, followed by 40 cycles of 95 ℃ for 10 s and 60 ℃ for 30 s. Fluorescence signals were acquired at the end of each cycle.

### LFD assembly and preparation

#### Synthesis and characterization of AuNPs and antibody conjugation

Colloidal gold nanoparticles were synthesized by the citrate reduction method. Briefly, 100 mL of 0.01% (w/v) HAuCl₄ in deionized water (254169, Sigma-Aldrich, St. Louis, MO, USA) was heated to boiling with vigorous stirring. Then, 2.5 mL of 1% (w/v) trisodium citrate (BF039Q, Heart Biological Technology Co., Ltd., Shanghai, China) was added rapidly. Upon stabilization of the solution to a purple-red color, it was cooled and diluted to the original volume with deionized water (100 mL), completing the preparation of AuNPs. The morphology and core diameter of AuNPs were characterized by transmission electron microscopy (TEM). For sample preparation, the gold nanoparticles were dispersed onto a 200-mesh copper grid coated with a carbon support film and negatively stained with phosphotungstic acid (PTA). The hydrodynamic diameter, particle size distribution, and polydispersity index of AuNPs were characterized by dynamic light scattering (DLS); all measurements were performed in triplicate.

The optimal amount of anti-6-FAM monoclonal antibody (Zhuhai Bomei Biotechnology Co., Ltd., Zhuhai, China) for AuNPs conjugation was determined by a salt flocculation assay. A volume of 125 µL of AuNP solution (pH adjusted to 8.5 with 0.1 M K₂CO₃) was mixed with 25 µL of serially diluted anti-6-FAM monoclonal antibody (0–20 µg/mL) and incubated at room temperature for 10 min. Subsequently, 100 µL of 10% NaCl (Servicebio, GC102004-500 g, Wuhan, China) solution was added, followed by another 10-minute incubation. The minimum antibody concentration that prevented color change from red to blue was considered the stabilization concentration. During the scaling-up preparation, 120% of this concentration was used to ensure sufficient antibody binding to the AuNPs.

#### Conjugate pad preparation and test strip assembly

**Fig. 2:**
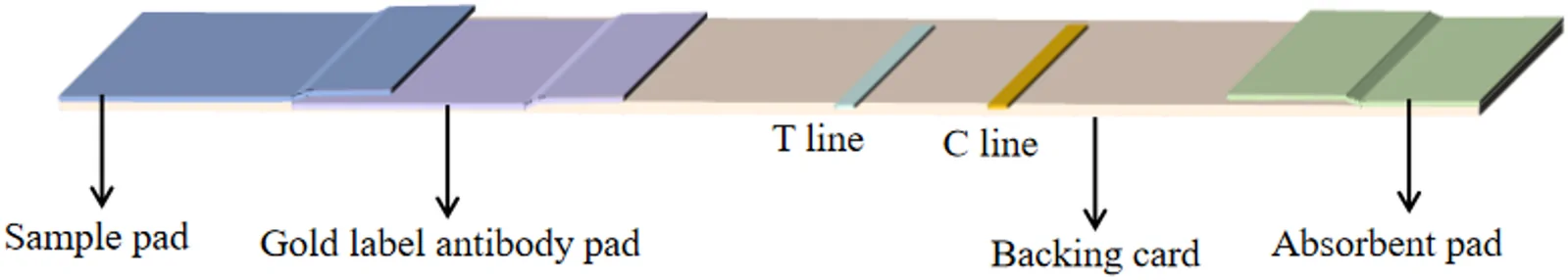
Schematic diagram of the LFD assembly process. The device consists of four key components assembled on a backing card: a sample pad for loading the sample, a conjugate pad (gold-labeled antibody pad) pre-loaded with gold nanoparticle-labeled detection antibodies, an NC membrane with immobilized test line and control line, and an absorbent pad to drive capillary flow

The anti-6-FAM monoclonal antibody-conjugated AuNPs were centrifuged at 12,000 g for 20 min at 4 °C. The pellet was subsequently resuspended in preservation buffer composed of 10 mM PBS (pH 7.4; G4250-500mL, Servicebio, Wuhan, China) supplemented with 1% BSA (V900933, Sigma-Aldrich, St. Louis, MO, USA) and 0.1% Tween-20 (GC204002-100 mL, Servicebio, Wuhan, China). For strip assembly, the nitrocellulose (NC) membrane (10–12 μm) was laminated onto a PVC backing card. Subsequently, the treated conjugate pad, sample pad, and absorbent pad were sequentially affixed to the card (all components obtained from Goldbio Tech Co., Ltd., Shanghai, China), ensuring a 2 mm overlap at each junction (Fig. 2). The assembled card was cut into 2.9 mm × 60 mm strips using a strip cutter and stored in resealable bags.

#### Assay procedure and readout

After RPA amplification, 10 µL of the amplified RPA product was diluted in 90 µL of PBS. The LFD was then inserted into this diluted solution. Results were recorded after 5 min of incubation. The presence of both red lines (C and T) indicated a positive result for the *gE* gene of wild-type PRV. The presence of only the C line indicated a negative result.

### Analytical sensitivity and specificity of RPA-LFD

The plasmid templates were serially diluted to obtain different concentration, and each dilution was tested three times using the RPA-LFD method. The visually limit of detection (LOD) was defined as the lowest concentration at which all replicates showed a clear T line. All diluted plasmid templates were also subjected to conventional PCR, qPCR, and agarose gel electrophoresis to evaluate the sensitivities of the RPA-LFD and conventional PCR and qPCR assays.

The plasmid templates were serially diluted to generate a series of concentration gradients, and each dilution was tested in triplicate using the RPA‑LFD assay. The visually detectable limit of detection (LOD) was defined as the lowest template concentration at which a clear T line was observed across all replicates. All serially diluted plasmid templates were analyzed by conventional PCR coupled with agarose gel electrophoresis to compare the detection sensitivity of the RPA‑LFD assay and the conventional amplification method. Meanwhile, qPCR was adopted as a reference method to verify the actual copy number of template samples and corroborate the reliability of the results obtained from the above two detection assays.

To evaluate the specificity of the RPA-LFD method, viral nucleic acids (DNA/RNA) were extracted from the cell culture supernatants of PRV, PRRSV, PCV-2, PEDV, TGEV, and CSFV using commercial kits. After RNA was reverse-transcribed into cDNA, RPA amplification was performed using the extracted DNA and the cDNA reverse-transcription products as templates. Each viral nucleic acid sample was subsequently analyzed by PCR (with agarose gel electrophoresis) and qPCR to verify the RPA-LFD results. In the RPA, conventional PCR, and qPCR amplification systems, PRV genomic DNA and DEPC-treated water were used as the positive and negative controls, respectively.

### Data analysis

To improve the objectivity of the detection results, ImageJ software was used to analyze the band intensities on the LFD strips. Histograms and statistical graphs were generated using Origin 2023 software.

## Results

### Characterization of colloidal gold particles and optimization of antibody labeling amount

**Fig. 3:**
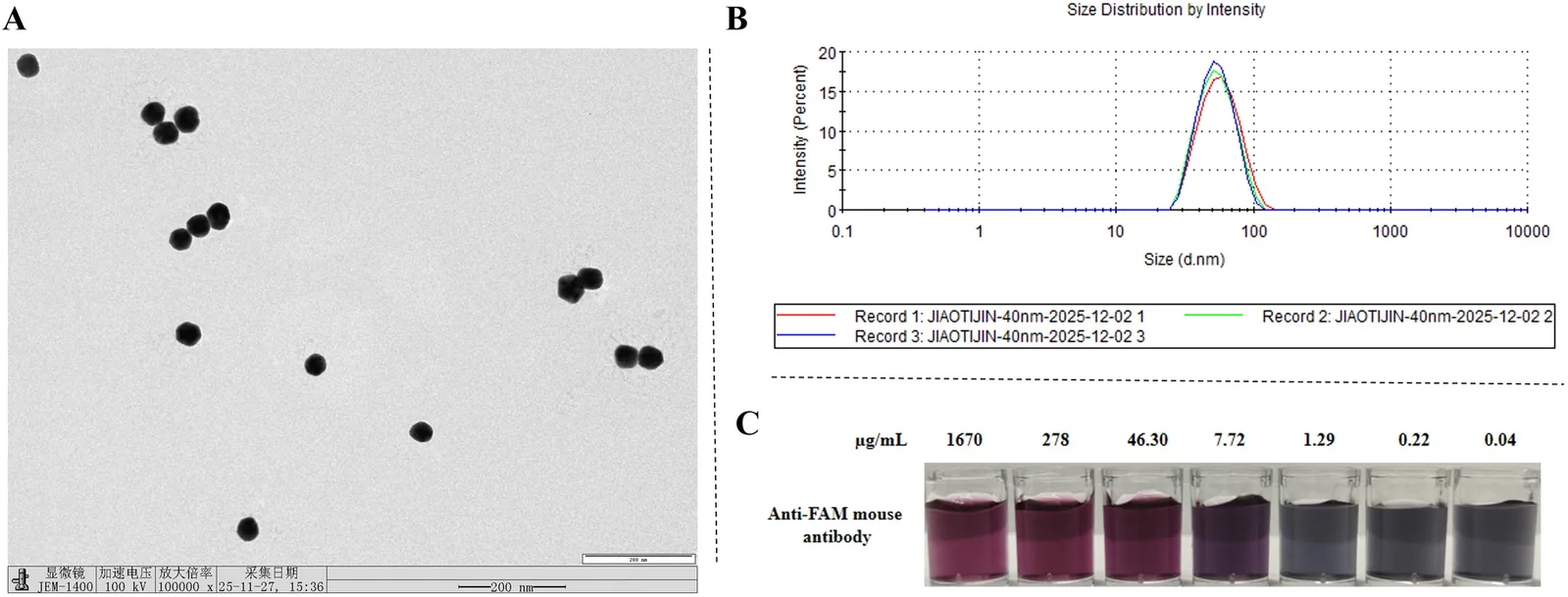
TEM and DLS analyses of AuNPs and optimization of anti-6-FAM monoclonal antibody labeling amount. **A** TEM characterization of the AuNPs. Scale bar = 200 nm. **B** DLS analysis of the AuNPs repeated three times. **C** The optimal labeling amount of 6-FAM monoclonal antibody. Red : well-dispersed AuNPs, successfully labeled; blue : aggregated due to insufficient labeling

The synthesized AuNPs solution appeared purplish-red. TEM analysis revealed well-dispersed, spherical nanoparticles with diameters ranging from 40 to 50 nm (Fig. 3A). DLS analysis indicated that the Z-average hydrodynamic diameter of these AuNPs was 47.79 nm (Fig. 3B). The intensity-weighted size distribution showed a unimodal profile, with the main peak at 58.95 nm. The polydispersity index obtained was 0.225, indicating a moderately broad size distribution. Notably, the monomodal distribution and absence of secondary peaks confirmed the sample system’s monocomponent nature, with no significant agglomerates or minor impurities detected.

Based on the salt flocculation assay, the minimum monoclonal antibody concentration that prevented colloidal gold precipitation and maintained a burgundy color was 46.30 µg/mL, which was established as the optimal labeling concentration (Fig. 3C). For subsequent preparations, a 20% excess (55.56 µg/mL) was used to ensure complete coating.

### Selection of RPA primers and probes

**Fig. 4:**
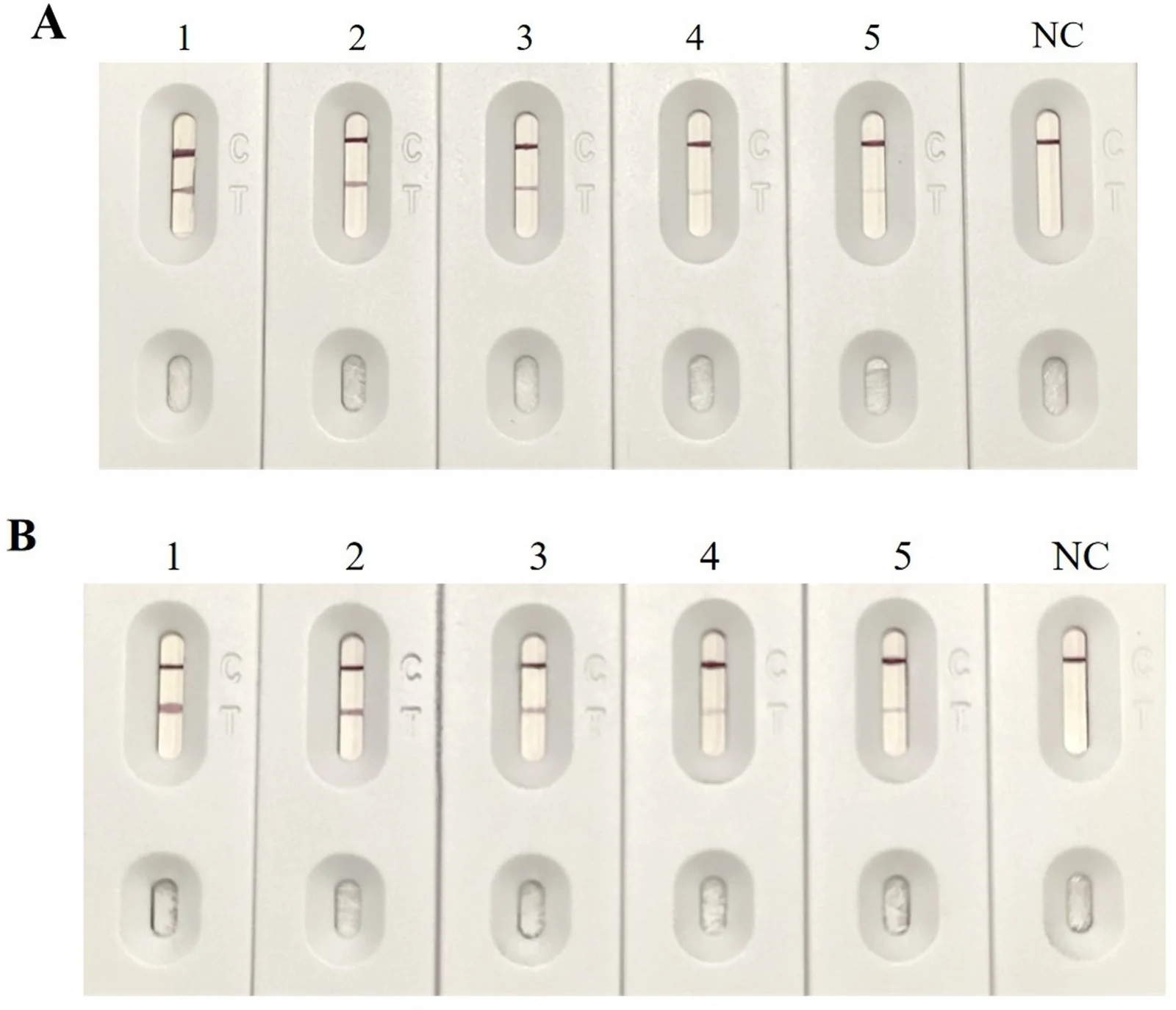
Optimization of RPA primers and probes. 1–5: 1:9, 1:49, 1:99, 1:199, and 1:399, respectively. **A** LFD results for RPA products amplified with the F1/R1/P1 primer-probe set after dilution with PBS. **B** LFD results for RPA products amplified with the F2/R2/P2 primer-probe set after dilution with PBS

The amplification efficiency of the primers and probes was evaluated through preliminary RPA-LFD experiments. An optimal primer-probe combination was determined by observing and comparing color development on LFDs after serial dilution of RPA reaction products. The results indicated no significant difference between the *gE*-F1/R1/P1 and gE-F2/R2/P2 combinations (Fig. 4Aand 4B). Therefore, the *gE*-F2/R2/P2 primer-probe set was selected and used to develop the RPA-LFD detection method.

### Optimization of RPA reaction conditions

**Fig. 5:**
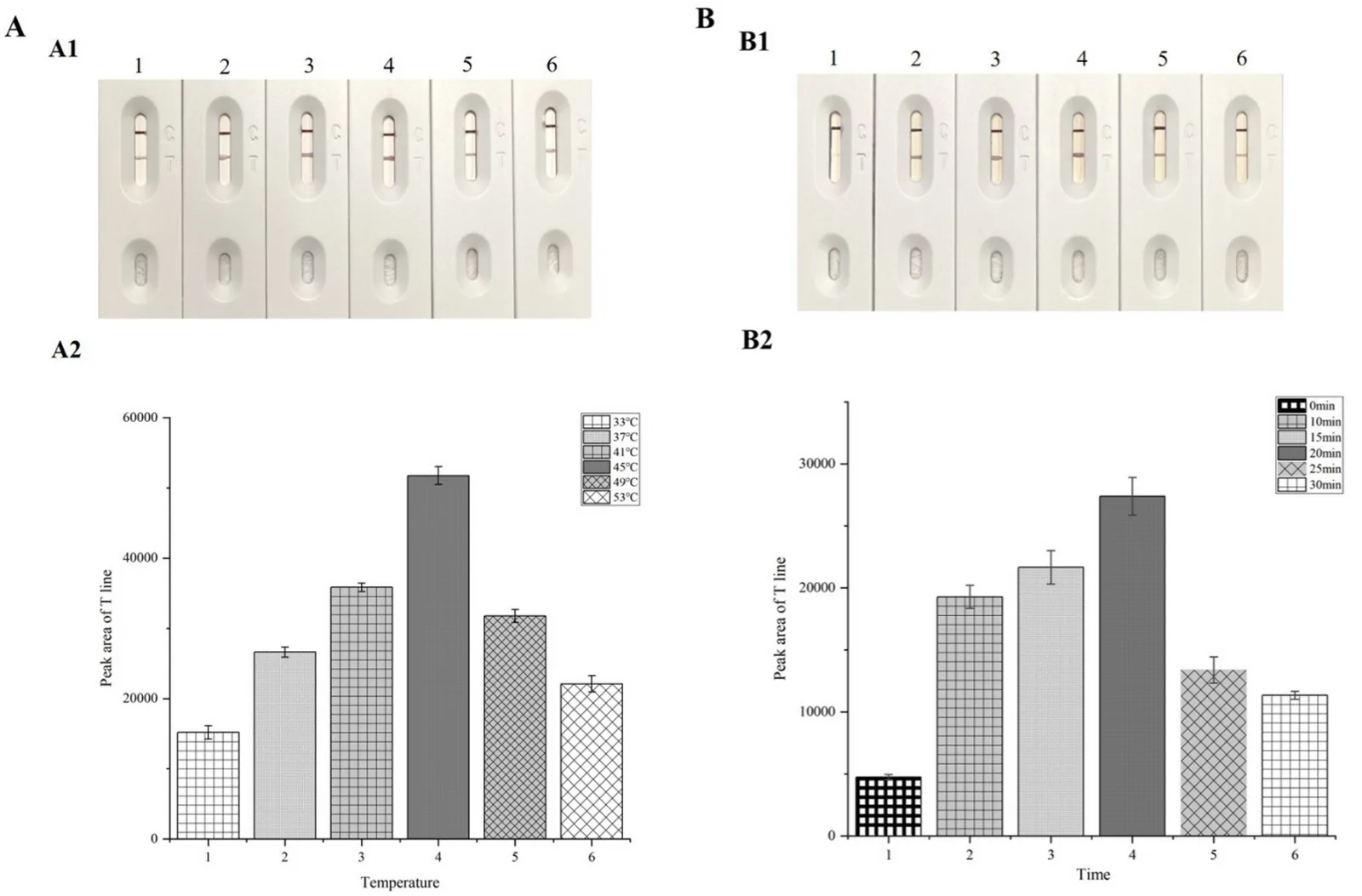
Optimization of RPA reaction temperature and time. **A** 1–6: 33℃, 37℃, 41 ℃, 45 ℃, 49 ℃, 53 ℃. A1: Results of temperature optimization for RPA-LFD. A2: Grayscale analysis of the T line intensity at various reaction temperatures. **B** 1–6: 0 min, 10 min, 15 min, 20 min, 25 min, 30 min. B1: Results of time optimization for RPA-LFD. B2: Grayscale analysis of the T line intensity at various reaction times

The amplification temperature and incubation time of the RPA reaction were optimized. Visual assessment and quantitative grayscale analysis (via ImageJ) of the T line intensity indicated that 45 °C was the optimal temperature, yielding the strongest signal (Fig. 5A1 and A2). Time-course experiments showed that a 20-minute reaction produced maximal T line intensity, with weaker or diminishing signals observed at shorter or longer durations (Fig. 5B1 and B2). Consequently, an amplification condition of 45 °C for 20 min was adopted as the standard RPA condition.

### Analytical sensitivity of RPA-LFD

**Fig. 6:**
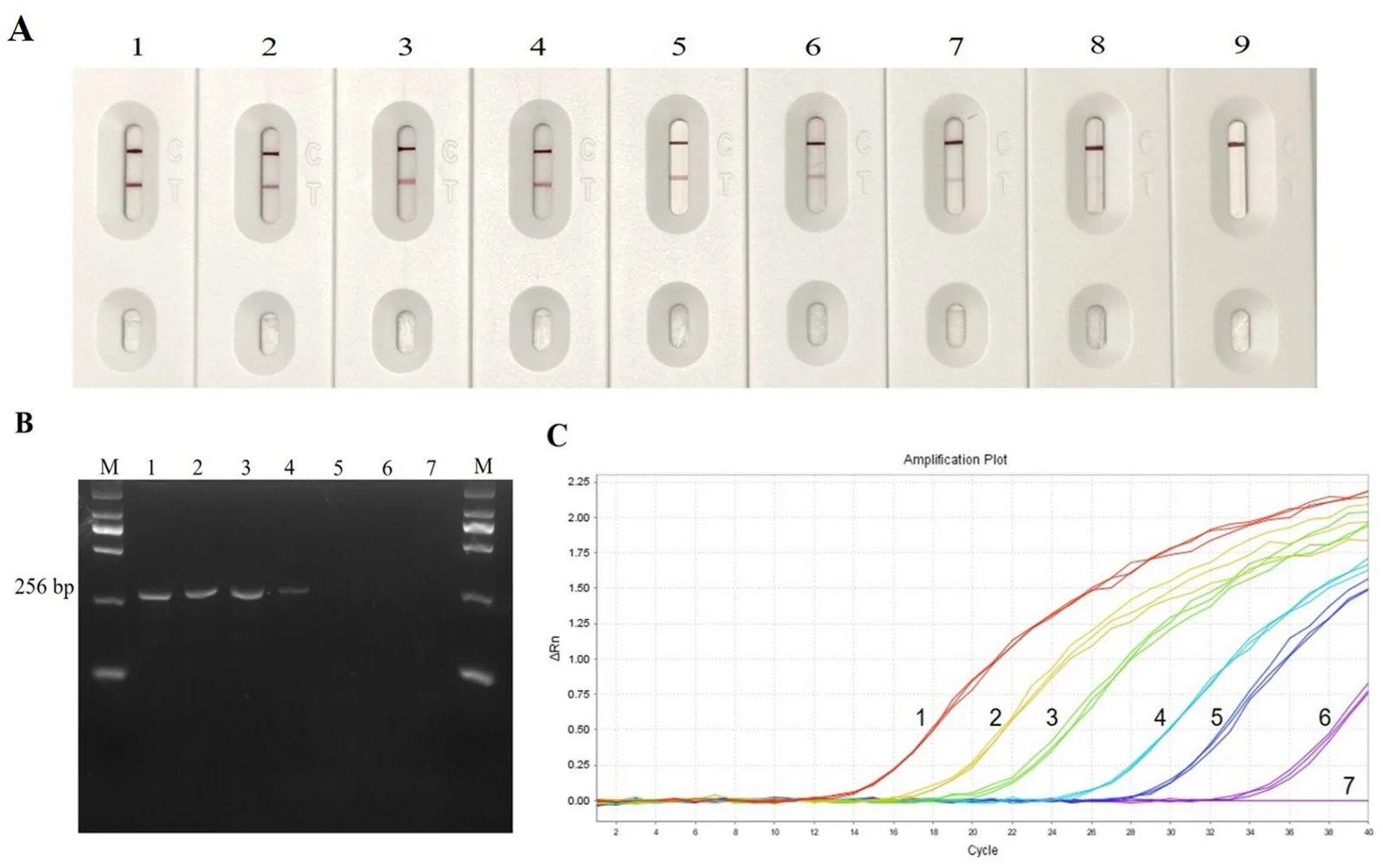
Sensitivity of RPA-LFD. **A** Eight serial dilutions of PRV gE templates were used to evaluate the limit of detection of the RPA-LFD assay. 1–9: 8.0 × 10⁷, 8.0 × 10⁶, 8.0 × 10⁵, 8.0 × 10⁴, 8.0 × 10³, 8.0 × 10², 8.0 × 10¹, 8.0 × 10⁰ copies/µL, and NC, respectively. **B** Six serial dilutions of PRV-gE templates were used to evaluate the limit of detection of the PCR assay. M: DL 2000 DNA marker; 1–7: 8.0 × 10⁷, 8.0 × 10⁶, 8.0 × 10⁵, 8.0 × 10⁴, 8.0 × 10³, 8.0 × 10² copies/µL, and NC, respectively. **C** Six serial dilutions of PRV-gE templates were used to evaluate the limit of detection of the qPCR. 1–7: 8.0 × 10^8^, 8.0 × 10^7^, 8.0 × 10^6^, 8.0 × 10^5^, 8.0 × 10^4^, 8.0 × 10^3^copies/µL, and NC, respectively

To assess the sensitivity of the RPA-LFD method, a 10-fold serial dilution of positive recombinant plasmids was used as the template. The RPA-LFD method achieved a LOD of 80 copies/µL for the *gE* gene, with clear red T lines visible in the concentration range of 8.0 × 10⁷ to 80 copies/µL (Fig. 6A), while the signal at 8 copies/µL showed no significant difference from the negative control. The LOD of conventional PCR was 8.0 × 10⁴ copies/µL (Fig. 6B). As a reference calibration tool in this study, qPCR accurately quantified the actual template copy number and yielded a validated LOD of 8.0 × 10³ copies/µL (Fig. 6C). Based on the reliable quantitative results verified by qPCR, our data confirmed that the established RPA-LFD method exhibited markedly higher detection sensitivity than conventional PCR under identical experimental conditions.

### Analytical specificity of RPA-LFD

**Fig. 7:**
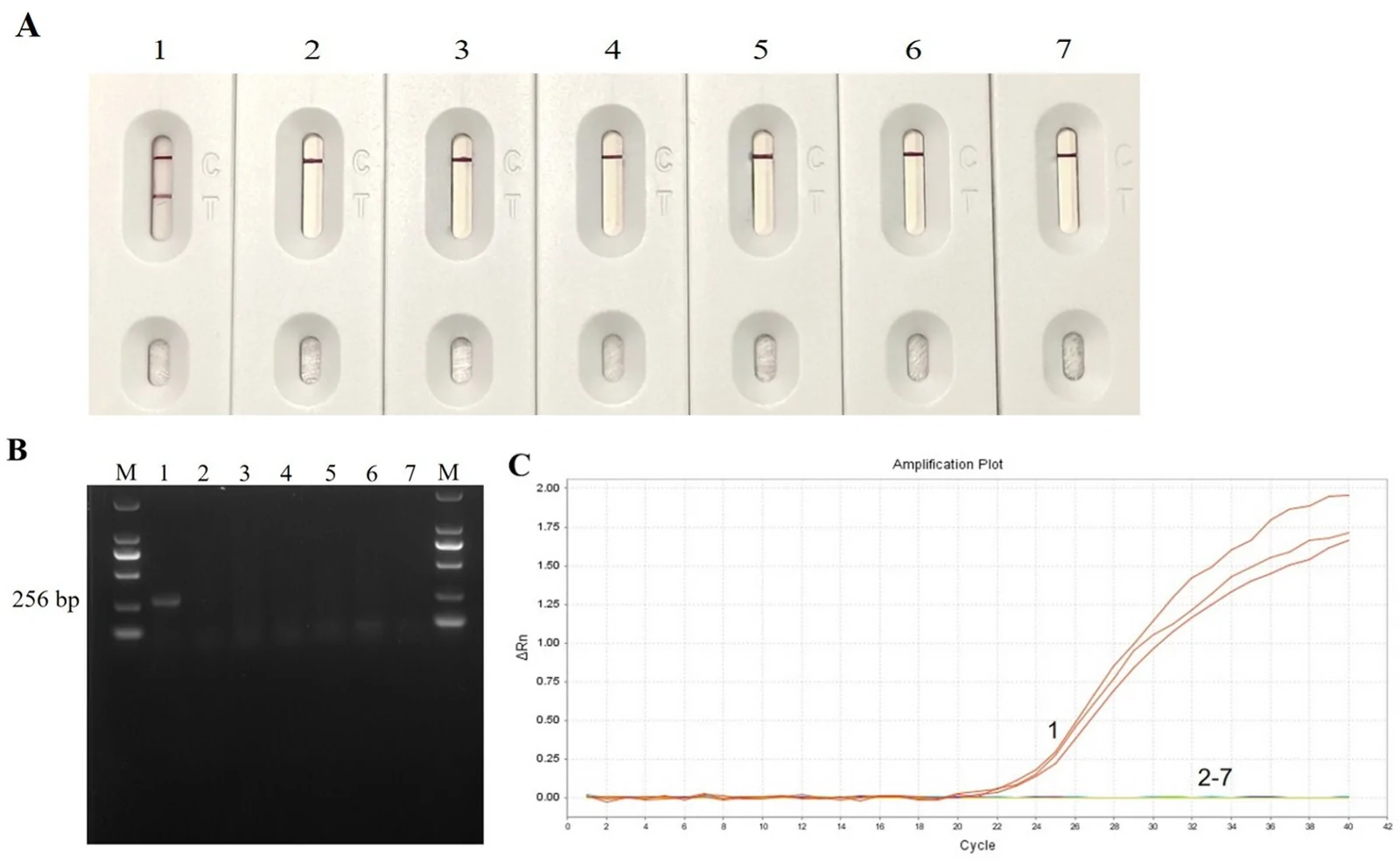
Specificity of RPA-LFD. M: DL 2000 DNA marker; 1–7: PRV, PRRSV, PCV-2, PEDV, TGEV, CSFV, and NC, respectively. **A** RPA-LFD detection results for different viral templates. **B** Detection results of the PCR assay for different viral templates. **C **Detection results of qPCR assay for different viral templates

The specificity of the established method was evaluated using genomic DNA or cDNA derived from common swine pathogens, including PRRSV, PCV-2, PEDV, TGEV, and CSFV. As shown in Fig. 7A, the method exhibited excellent specificity: only the wild-type PRV (*gE*-positive) sample produced clear T and C lines, with no cross-reactivity observed with the other swine pathogens mentioned above. Electrophoresis of the PCR products and qPCR results showed that amplification was only achieved using PRV genomic DNA as the template (Fig. 7B and C). These results indicate that the established RPA-LFD assay has high specificity and reliability.

## Discussion

The (RT-)RPA-LFD method offers rapid detection, high sensitivity, specificity, and field suitability, and has been successfully applied to multiple important swine pathogens with robust performance. For instance, an RT-RPA-LFD assay targeting the PEDV N gene achieved a detection limit of 10² copies/µL in 30 min at 37 °C with no cross-reactivity with other common swine pathogens. Similarly, an RPA-LFD assay for the PRV gD gene achieved detection within 20 min at 39 °C. Furthermore, an RPA-LFD assay for detecting MGF-505R gene-deleted mutants of African swine fever virus reached a detection limit of 10 copies/µL in 20 min at 37 °C, demonstrating high sensitivity and specificity [27–29].

PRV belongs to the a *Alphaherpesvirus* subfamily and primarily infects pigs, causing significant economic losses to the global swine industry [30]. *gE*-deleted strains have long been used in vaccine research, and *gE* can serve as a diagnostic marker to differentiate between vaccinated and wild-type virus-infected pigs [31]. This study established an RPA-LFD detection system targeting the *gE* gene, enabling rapid detection and differentiation of wild-type PRV from *gE*-deleted vaccine strains. This method requires no specialized equipment, achieves a sensitivity of 80 copies/µL, and exhibits excellent specificity, with no cross-reactivity with other major swine viral pathogens.

Compared with conventional PCR and qPCR, the newly established method in this study not only exhibits superior sensitivity but also confers substantial advantages across multiple key dimensions: First, conventional PCR and qPCR typically require a run time of 1–1.5 h, whereas the full RPA-LFD workflow can be completed within 25 min, comprising a 20-min isothermal amplification followed by a 5-min visual readout via lateral flow strips. Such a rapid turnaround enables same-day diagnostic decision-making, thereby mitigating economic losses stemming from delayed pathogen isolation or missed intervention windows. Second, PCR-based methods rely on thermal cyclers for precise temperature cycling control. In contrast, the RPA amplification described herein proceeds isothermally at 45 °C, and is theoretically compatible with a broad temperature range from 25 °C to 45 °C. The subsequent LFD readout is performed at ambient temperature. Third, PCR-based approaches require dedicated instrumentation and controlled laboratory settings, while RPA-LFD results are directly interpretable by the naked eye, eliminating the need for specialized technical personnel and dedicated PCR facilities. This markedly reduces indirect costs associated with personnel training and infrastructure maintenance.

In brief, the direct reagent cost of a single RPA‑LFD reaction is slightly higher than that of conventional PCR and comparable to that of qPCR under our experimental conditions. Nevertheless, RPA‑LFD eliminates the reliance on costly thermal cyclers, real‑time fluorescence instruments, agarose gel electrophoresis, and imaging systems, and requires substantially shorter hands‑on operation time. When equipment depreciation and labor expenditure are comprehensively considered, the platform possesses prominent advantages for point‑of‑care and field testing in resource‑limited settings.

Although this method can rapidly and accurately detect wild-type PRV infection and differentiate it from vaccination with *gE*-deleted vaccine strains, it still has certain inherent methodological limitations. For instance, the test results rely on visual interpretation, which may carry a risk of false-positive judgments.

Within the DIVA-based test-and-removal framework for PRV eradication, our RPA-LFD field assay not only supports timely culling of PRV-positive animals by providing rapid diagnostic evidence, but also enables routine herd surveillance for early detection of latent infections and interruption of silent virus transmission. It facilitates on-site quarantine during animal introduction and cross-farm movement to prevent virus carriers from entering naive herds. During suspected outbreaks, it allows rapid epidemiological screening to delineate infection foci and guide isolation and control measures. Additionally, it supports on-site evaluation of PRV eradication efficacy via high-frequency sampling to confirm herd negativity.

Without requiring sophisticated laboratory equipment and with a low technical threshold, this assay can be deployed directly on farms, markedly shortening diagnostic turnaround time and narrowing the virus transmission window. It also enables preliminary screening of large sample batches to rule out negative specimens, with only suspicious samples referred for confirmatory laboratory qPCR, thereby reducing labor and reagent costs. Collectively, this method provides a practical on-site tool for PRV prevention and eradication under the DIVA strategy [32, 33].

## Conclusion

We have established a rapid, visual, and equipment-minial molecular assay for distinguishing wild-type PRV from vaccine strains. This assay meets the critical need for decentralized diagnostic capacity in pseudorabies control programs. Future studies will focus on validating its performance on diverse clinical samples (e.g., serum, tissue homogenates, and oral fluids) and exploring multiplexing capabilities for simultaneous detection of additional swine pathogens.

## Supplementary Information

Supplementary Material 1.

## Data Availability

The data supporting the findings of this study (PRV *gE* DNA sequences) are publicly available in the NCBI GenBank database. The accession numbers for the sequences used in this study are: OR062216.1.
